# A Moments-Based Analytical Approach for Cell Size Homeostasis

**DOI:** 10.1109/lcsys.2024.3411041

**Published:** 2024-06-07

**Authors:** César Nieto, Cesar Augusto Vargas-Garcia, Abhyudai Singh

**Affiliations:** Department of Electrical and Computer Engineering, University of Delaware, Newark, DE 19716 USA; AGROSAVIA - Corporacion Colombiana de Investigacion Agropecuaria, Mosquera, Cundinamarca 250047, Colombia; Department of Electrical and Computer Engineering, Biomedical Engineering, Mathematical Sciences, Center of Bioinformatics and Computational Biology, University of Delaware, Newark, DE 19716 USA

**Keywords:** Systems biology, stochastic systems, hybrid systems

## Abstract

This contribution explores mechanisms that regulate the dynamics of single-cell size, maintaining equilibrium around a target set point. Using the formalism of Stochastic Hybrid Systems (SHS), we consider continuous exponential growth in cell size (as determined by volume/mass/surface area). This continuous-time evolution is interspersed by cell division events that occur randomly as per a given size-dependent rate, and upon division, only one of the two daughter cells is tracked. We show that a size-independent division rate does not provide cell size homeostasis, in the sense that the variance in cell size increases unboundedly over time. Next, we consider a division rate proportional to cell size that yields the *adder* size control observed in several bacteria – a constant size is added on average between birth and division regardless of the newborn size. For this scenario, we obtain exact formulas for the steady-state moments (mean, variance, and skewness) of cell size. Expanding the SHS model, we explore a biologically relevant scenario where the time between successive division events is further divided into multiple discrete stages with size-dependent stage transitions. Exact moment computations demonstrate that increasing the number of stages reduces cell size variability (noise). We also find formulas considering uneven size partitioning between daughters during division, and where the division rate follows a power law of the cell size leading to deviations from adder size control. This letter provides a method for estimating model parameters from observed cell size distributions and enhances our understanding of mechanisms underlying cell size regulation.

## I. Introduction

Single cells proliferate by growing in size over time, followed by subsequent division into newborn daughters. Cell size is homeostatically regulated around a setpoint by mitigating errors incurred during these growth-division cycles [1], [2], [3]. Cell size control has been phenomenologically characterized by adder and sizer strategies. Adder control implies a fixed cell size added on average during the cell cycle from birth to division. These adders have been reported for diverse microbes [1], [3], [4], [5], and also in human cells [6], [7]. In contrast, sizer control, as observed in fission yeast [8], implies cell division occurring when cell size reaches a prescribed size threshold [9].

Cell size dynamics has previously been modeled using the framework of Stochastic Hybrid Systems (SHS) integrating continuous growth in cell size with discrete random division events that occur at a size-dependent rate [10], [11], [12], [13]. Division events reset the cell size to a half allowing for an arbitrary level of error in this size partitioning. Our prior work characterized the critical requirements on both the cellular growth rate and division rate needed for cell size homeostasis. For example, coupling exponential growth in cell size with size-independent division rate is nonhomeostatic, leading to unbounded growth in the intercellular size variance over time [10]. Active size control results in a bounded variance and the *adder* requires a division rate proportional to cell size [14], [15].

Here we provide formulas for the lower-order moments of cell size when the division rate increases with cell size s as $s^{\alpha }$ (where α>0). Specifically, for α=1, we observe the *adder* strategy, and for α→∞, we encounter the *sizer*. Our results are exact for the adder and approximate for α>1 but validated through stochastic simulations. We also consider a more realistic scenario for the adder, where the time between two consecutive divisions is split into multiple stages, with size-dependent transitions between them. We obtain an exact formula for the steady-state mean and noise in cell size for this multi-stage model and use it to determine the fundamental limit of reducing cell size variability.

### Notation:

The angular brackets notation 〈〉 denote the expected value of random variables and random processes. An overline on the angular brackets, $\bar{\langle \;\rangle }$ represents the expected value of random processes as t→∞. We use log to represent the natural logarithm. $CV_{x}$ denotes the coefficient of variation used to quantify the random variability (noise) of a random variable x.

## II. SHS Framework for Cell Size Dynamics

Let the positive real-valued variable s(t) denote the size of an individual cell at time t≥0. Assuming exponential growth in cell size, s(t) evolves according to the ordinary differential equation:

(1) $$\frac{ds}{dt}=\mu s$$

where constant μ>0 is known as the fractional *growth rate*. Cell division events occur randomly, with a division rate that scales according to the power law of cell size: $ks^{\alpha }$, where k and α are non-negative constants. To account for stochasticity in division timing, we consider the probability that a cell division event is triggered within the next infinitesimal time interval (t,t+dt] to be $ks^{\alpha }dt$. The exponent α also reflects the log-sensitivity of the division rate to the cell size. In particular, when α>0, the exponential growth of s(t) leads to division events following a non-homogeneous Poisson process [10]. Moreover, when α=0, the division rate is constant k, resulting in the classical Poisson process timing.

**Fig. 1.**
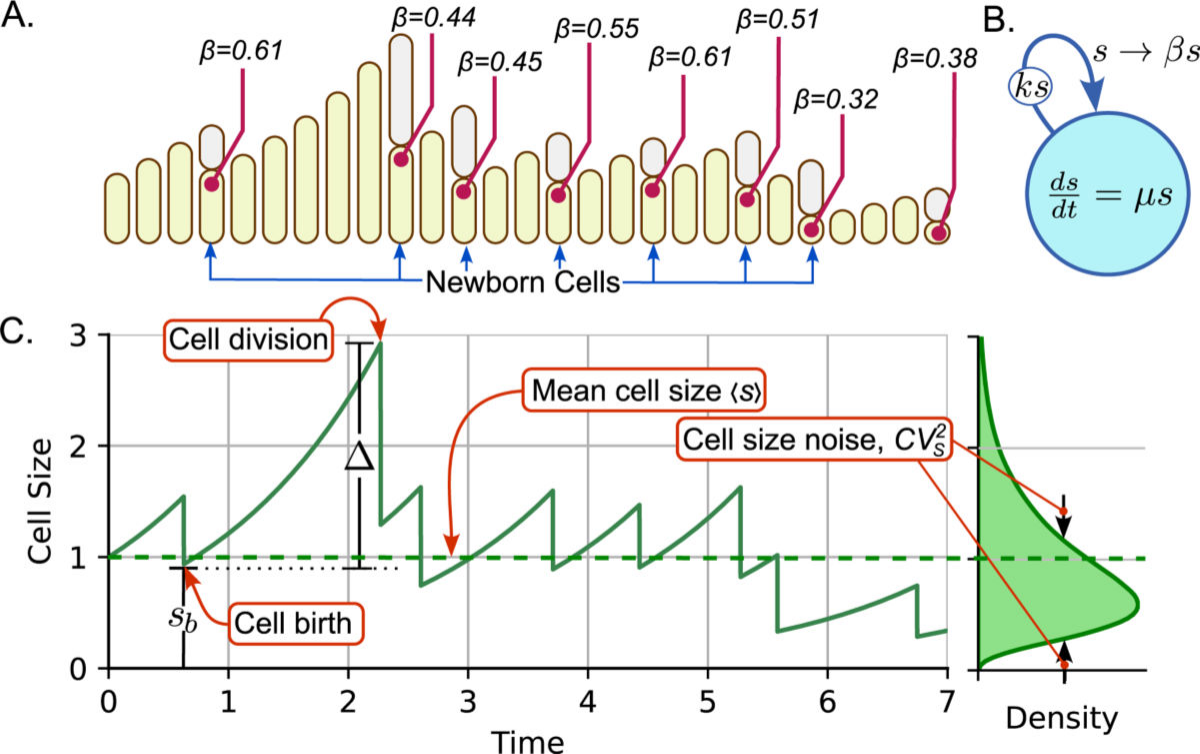
A) Cell growth and division with randomness in size partitioning during division quantified by β. After division, one cell is discarded (gray cells). B) SHS model schematic where cell size grows continuously according to the differential equation within the circle. The arrows represent division events that occur with rate ks (node on the arrow), and the cell size reset (division by half with some randomness captured by β) is shown at the end of the arrow. C) (left) Cell size over time showing the size at birth $s_{b}$ and added size Δ within a cell cycle. (right) The cell size distribution estimated from multiple realizations of cell size trajectories.

As shown in Fig. 1A, when a division event is triggered, the parent cell divides into two daughters. Following the SHS formalism, we keep one of the descendants and discard the other one [10]. Division is modeled as a reset (Fig. 1B) where the cell size is approximately halved. The overall SHS model is represented as

(2) $$\frac{ds}{dt}=\mu s,\;\text{for}\;t\geq 0,\;\text{except}\;t=t^{*}\;s^{+}=\beta s,\;\text{for}\;t=t^{*}$$

where $t^{*}$ accounts for the time instant of division and $s^{+}$ is the size just after the reset. Here β∈(0,1) is a random variable with mean 〈β〉, for simplicity, modeled by a beta distribution [16]. For a perfect symmetric split of the parent cell, β=1/2 with probability one. Errors in the splitting position can be captured by noise in β that is quantified by $CV_{\beta }^{2}$ – the squared coefficient of variation of β defined as

(3) $$CV_{\beta }^{2}:=\frac{\langle \beta^{2}\rangle -{\langle \beta \rangle }^{2}}{{\langle \beta \rangle }^{2}}.$$

Scenarios where 〈β〉≠1/2 are also possible. For example, if a parent cell divides asymmetrically and the larger newborn is always chosen, then 〈β〉>1/2. However, if the newborn is chosen randomly, then 〈β〉=1/2, and the asymmetry in the division would be reflected in a higher $CV_{\beta }^{2}$.

Having formulated the stochastic dynamical system, our goal is to determine the following steady-state statistical moments of cell size of order p

(4) $$\bar{\langle s^{p}\rangle }:=\underset{t\to \infty }{\lim}\langle s{\left(t\right)}^{p}\rangle ,\;p\in \{1,2,\ldots \}.$$

## III. Quantifying Statistical Moments in Single-Step Models

To write the moment dynamics of cell size, we use the following result that allows computations of expectation of SHS state space: the time evolution of the expected value of any continuously differentiable function f(s) is given by

(5) $$\frac{d\langle f(s)\rangle }{dt}=\langle \mu s\frac{df}{ds}+\int_{y=0}^{1}ks^{\alpha }(f(ys)-f(s))p_{\beta }(y)dy\rangle$$

where $p_{\beta }$ is the probability density function of β∈(0,1) [17]. The first term on the right-hand side of (5) is the contribution from the SHS continuous dynamics, while the second term resulting from discrete stochastic events is a product of two terms: the rate of event occurrence and the change in f(s) when the event is triggered. We start with the simplest case of a size-independent division rate (α=0).

### A. Size-Independent Division Rate

Setting α=0 and f(s)=s in (5) results in

(6) $$\frac{d\langle s\rangle }{dt}=\langle \mu s+k\left(s\int_{y=0}^{1}yp_{\beta }(y)dy-s\right)\rangle \;\;=\mu \langle s\rangle -k(1-\langle \beta \rangle )\langle s\rangle .$$

To have a non-zero bounded mean cell size, this case requires the division rate to exactly balance the growth rate k=μ/(1−〈β〉). Now deriving the dynamics of the second-order (uncentered) moment

(7) $$\frac{d\langle s^{2}\rangle }{dt}=2\mu \langle s^{2}\rangle -k\left(1-\langle \beta^{2}\rangle \right)\langle s^{2}\rangle$$

and setting k=μ/(1−〈β〉) result in

(8) $$\frac{d\langle s^{2}\rangle }{dt}=\mu \langle s^{2}\rangle \left(2-\frac{1-\langle \beta^{2}\rangle }{1-\langle \beta \rangle }\right)\;\;=\mu \langle s^{2}\rangle \left(\frac{{\left(1-\langle \beta \rangle \right)}^{2}+\langle \beta^{2}\rangle -{\langle \beta \rangle }^{2}}{1-\langle \beta \rangle }\right).$$

Since 〈β〉<1 and $\langle \beta^{2}\rangle \geq {\langle \beta \rangle }^{2}$,

(9) $$\frac{d\langle s^{2}\rangle }{dt}=\mu \langle s^{2}\rangle \left(\frac{{\left(1-\langle \beta \rangle \right)}^{2}+\langle \beta^{2}\rangle -{\langle \beta \rangle }^{2}}{1-\langle \beta \rangle }\right)>0$$

(10) $$\Rightarrow \underset{t\to \infty }{\lim}\langle s{\left(t\right)}^{2}\rangle =\infty .$$

Thus, the variance in cell size grows unbounded over time. In this context, cell division events follow a Poisson process with a constant rate k, corresponding to inter-division times (i.e., cell-cycle times) drawn from an exponential distribution with mean 1/k. Results for any arbitrary size-independent cell cycle times have been addressed in previous work [10]. The main conclusion is that, in a size-independent cell cycle timing, even the slightest noise in the system leads to unbounded growth in $\langle s^{2}\rangle$. Consequently, *active size sensing* (triggering division events based on cell size) is a *necessary feature of cell size homeostasis* for cells that grow exponentially.

### B. Adder Control of Cell Size

Next, we aim to estimate the cell size moments for the case of α=1 with division events occurring at a rate ks proportional to cell size s. This division rate implements the *adder*, where a cell born with size $s_{b}$ divides after adding a cell size Δ (Fig. 1C). With this rate, Δ is an independent and identically distributed random variable following an exponential distribution with mean μ/k [18].

To estimate the mean cell size, we write the moment dynamics of f(s)=logs obtaining

(11) $$\frac{d\langle \log(s)\rangle }{dt}=\mu +\langle ks(\log(\beta s)-\log(s))\rangle \;\;=\mu +k\langle s\rangle \langle \log(\beta )\rangle .$$

Solving this equation at steady-state yields the following mean cell size

(12) $$\bar{\langle s\rangle }=-\frac{\mu }{k\langle \log(\beta )\rangle }.$$

Similarly writing the moment dynamics of f(s)=s and solving at steady-state yields

(13) $$\;\frac{d\langle s\rangle }{dt}=\mu \langle s\rangle -k(1-\langle \beta \rangle )\langle s^{2}\rangle \;\Rightarrow \bar{\langle s^{2}\rangle }=\frac{\mu \bar{\langle s\rangle }}{k(1-\langle \beta \rangle )}.$$

Performing this process iteratively for $f(s)=s^{p}$ leads to exact steady-state moments

(14a) $$\bar{\langle s^{p+1}\rangle }=\frac{p\mu \bar{\langle s^{p}\rangle }}{k\left(1-\langle \beta^{p}\rangle \right)},\;p\in \{1,2,\ldots \}$$

(14b) $$=-{\left(\frac{\mu }{k}\right)}^{p+1}\frac{p!}{\langle \log(\beta )\rangle {\text{Π}}_{i=1}^{p}\left(1-\langle \beta^{i}\rangle \right)}.$$

From (12) and (13), the steady-state noise in cell size (as quantified by the squared coefficient of variation $CV_{s}^{2}$) is determined as

(15) $$CV_{s}^{2}:=\frac{\bar{\langle s^{2}\rangle }-{\bar{\langle s\rangle }}^{2}}{{\bar{\langle s\rangle }}^{2}}=-1+\frac{\langle \log(\beta )\rangle }{\langle \beta \rangle -1}$$

and is independent of both the growth rate μ and k. Note that since β∈(0,1)

(16) $$\frac{\langle \log(\beta )\rangle }{\langle \beta \rangle -1}=\frac{\langle \log(1/\beta )\rangle }{1-\langle \beta \rangle }>1$$

ensuring $CV_{s}^{2}>0$. For perfect partitioning (β=1/2 with probability one) we obtain:

(17) $$CV_{s}^{2}=-1+\frac{\log(1/2)}{1/2-1}=\log(4)-1\approx 0.39.$$

To study the effect of size partitioning errors during cell division we assume that β is a beta-distributed random variable with mean 〈β〉 and squared coefficient of variation $CV_{\beta }^{2}$, and in this case the expectation of log is given by:

(18) $$\langle \log(\beta )\rangle =\psi (z)-\psi (z/\langle \beta \rangle ),\;z=\frac{1-\langle \beta \rangle \left(1+CV_{\beta }^{2}\right)}{CV_{\beta }^{2}}$$

where ψ(z) is the digamma function [19] and can be computed in Wolfram Mathematica using the command *PolyGamma[z]*. The beta distribution is characterized by two shape parameters which will be equal when 〈β〉=1/2, and their sum is given by z/〈β〉. It is possible to obtain simpler formulas using the approximation,

(19) $$\psi (z)\approx \log(z)-\frac{1}{2z}$$

valid for z→∞ (which holds from (18) when $CV_{\beta }^{2}\ll 1$). Therefore, it is possible to approximate from (18)

(20) $$\langle \log(\beta )\rangle \approx \log(\langle \beta \rangle )-\frac{(1-\langle \beta \rangle )CV_{\beta }^{2}}{2\left(1-\langle \beta \rangle -\langle \beta \rangle CV_{\beta }^{2}\right)},\;CV_{\beta }^{2}\ll 1.$$

Using (20) in (15) we develop the following approximation.

(21) $$CV_{s}^{2}\approx -1+\left(\log(4)+\frac{CV_{\beta }^{2}}{1-CV_{\beta }^{2}}\right),\;CV_{\beta }^{2}\ll 1$$

valid for small partitioning errors around 〈β〉=1/2, where $CV_{s}^{2}$ is a monotonically increasing function of $CV_{\beta }^{2}$.

It is worthwhile to compare the noise in cell size (21) with the noise in newborn cell size $CV_{s_{b}}^{2}$, derived in previous research [16] and give

(22) $$CV_{s_{b}}^{2}=\frac{4CV_{\beta }^{2}+CV_{\text{Δ}}^{2}\left(1+CV_{\beta }^{2}\right)}{3-CV_{\beta }^{2}}$$

where $CV_{\text{Δ}}^{2}$ is the squared coefficient of variation of the added size Δ. In this model, added size is exponentially distributed $\left(CV_{\text{Δ}}=1\right)$ [14] and therefore

(23) $$CV_{s_{b}}^{2}=\frac{1+5CV_{\beta }^{2}}{3-CV_{\beta }^{2}}.$$

For perfect partitioning $\left(CV_{\beta }^{2}=0\right)$, $CV_{s_{b}}^{2}=1/3$. Note that $CV_{s_{b}}^{2}$ is more sensitive to $CV_{\beta }^{2}$ than $CV_{s}^{2}$ when partitioning errors are small, as can be seen by comparing the sensitivities:

(24) $$\frac{dCV_{s}^{2}}{dCV_{\beta }^{2}}{|}_{CV_{\beta }^{2}=0}=1,\;\frac{dCV_{s_{b}}^{2}}{dCV_{\beta }^{2}}{|}_{CV_{\beta }^{2}=0}=16/9.$$

This implies that for low $CV_{\beta }^{2}$, we have $CV_{s_{b}}^{2}<CV_{s}^{2}$ and for large $CV_{\beta }^{2}$, $CV_{s_{b}}^{2}>CV_{s}^{2}$.

It is also possible to explore higher moments. For instance, using the expression for the third-order uncentered moment from (14a), this moment satisfies

(25) $$\;\bar{\langle s^{3}\rangle }=\frac{2\mu \bar{\langle s^{2}\rangle }}{k\left(1-\langle \beta^{2}\rangle \right)}\;\Rightarrow \frac{\bar{\langle s^{3}\rangle }}{{\bar{\langle s\rangle }}^{3}}=-\frac{2\left(1+CV_{s}^{2}\right)\langle \log(\beta )\rangle }{\left(1-\langle \beta^{2}\rangle \right)}$$

an exact analytical expression can be derived for the steady-state skewness of cell size

(26) $$\gamma_{s}:=\frac{\frac{\bar{\langle s^{3}\rangle }}{{\bar{\langle s\rangle }}^{3}}-CV_{s}^{2}-1}{CV_{s}^{3}}.$$

This skewness remains roughly constant at $\gamma_{s}\approx 1.68$ for $0\leq CV_{\beta }^{2}<0.1$, and then increases with $CV_{\beta }^{2}$.

### C. Sizer Control of Cell Size

We now consider the case of a general division rate with any arbitrary exponent α>0. The case of α>1 has been described in the literature as a sizer-like control [14], [15], [16], with α→∞ corresponding to a division that occurs when the cell size reaches a threshold $k^{1/\alpha }$. If we define a new variable $y=s^{\alpha }$, then the stochastic dynamics of y can be recast as an SHS with continuous dynamics and reset

(27) $$\frac{dy}{dt}=\alpha \mu y,\;y\mapsto \beta^{\alpha }y$$

with resets occuring at a rate ky proportional to y. Thus, all cell size properties from the previous section now apply to y including the size-invariant adder: the increase in y within one cell cycle from birth to division will be drawn from an exponential distribution with mean αμ/k. Steady-state moments of y can be straightforwardly obtained as

(28) $$\bar{\langle y\rangle }=-\frac{\mu }{k\langle \log(\beta )\rangle }$$

(29) $$\bar{\langle y^{p+1}\rangle }=-{\left(\frac{\alpha \mu }{k}\right)}^{p+1}\frac{p!}{\alpha \langle \log(\beta )\rangle {\text{Π}}_{i=1}^{p}\left(1-\langle \beta^{\alpha i}\rangle \right)}$$

p∈{1,2,…}, by replacing μ and β in (12)-(14b) by αμ and $\beta^{\alpha }$, respectively,

A key question of interest is how the exponent α impacts the noise in cell size. The moment dynamics of log(s) and s evolve as

(30) $$\frac{d\langle \log(s)\rangle }{dt}=\mu +k\langle s^{\alpha }\rangle \langle \log(\beta )\rangle \;\;\frac{d\langle s\rangle }{dt}=\mu \langle s\rangle -k(1-\langle \beta \rangle )\langle s^{\alpha +1}\rangle$$

respectively, resulting in the steady-state of monomials of s:

(31) $$\bar{\langle s^{\alpha }\rangle }=-\frac{\mu }{k\langle \log(\beta )\rangle },\;\bar{\langle s^{\alpha +1}\rangle }=\frac{\mu \bar{\langle s\rangle }}{k(1-\langle \beta \rangle )}$$

at steady state. When α<1 exact solutions of $\bar{\langle s^{2}\rangle }$ and $\bar{\langle s\rangle }$ are possible. For example, if α=1/3 then $y=s^{1/3}$, and using (28) one can obtain

(32) $$\bar{\langle s\rangle }=\bar{\langle y^{3}\rangle },\;\bar{\langle s^{2}\rangle }=\bar{\langle y^{6}\rangle }.$$

When α>1, we solve the mean and noise in cell size using closure schemes that nonlinearly approximate high-order moments in terms of lower-order moments. To this end, we propose the use of the derivative matching closure scheme that is used when $CV_{s}^{2}<<1$ as in the log-normal distribution [12], [20]. This yields the following approximations:

(33) $$\bar{\langle s^{\alpha }\rangle }\approx \frac{{\bar{\langle s^{2}\rangle }}^{\frac{\alpha (\alpha -1)}{2}}}{{\bar{\langle s\rangle }}^{\alpha (\alpha -2)}},\;\bar{\langle s^{\alpha +1}\rangle }\approx \frac{{\bar{\langle s^{2}\rangle }}^{\frac{\alpha (\alpha +1)}{2}}}{{\bar{\langle s\rangle }}^{\alpha^{2}-1}}$$

which substituting in (31) and solving for $\bar{\langle s^{2}\rangle }$ and $\bar{\langle s\rangle }$ gives

(34a) $$\bar{\langle s\rangle }\approx {\left(\frac{\mu }{k}\right)}^{\frac{1}{\alpha }}{\left(\frac{\langle \beta \rangle -1}{\langle \log(\beta )\rangle }\right)}^{\frac{1+\alpha }{2\alpha }}\frac{1}{{\left(1-\langle \beta \rangle \right)}^{\frac{1}{\alpha }}}$$

(34b) $$CV_{s}^{2}\approx -1+{\left(\frac{\langle \log(\beta )\rangle }{\langle \beta \rangle -1}\right)}^{\frac{1}{\alpha }}$$

(34c) $$\approx -1+{\left(\log(4)+\frac{CV_{\beta }^{2}}{1-CV_{\beta }^{2}}\right)}^{\frac{1}{\alpha }}.$$

**Fig. 2.**
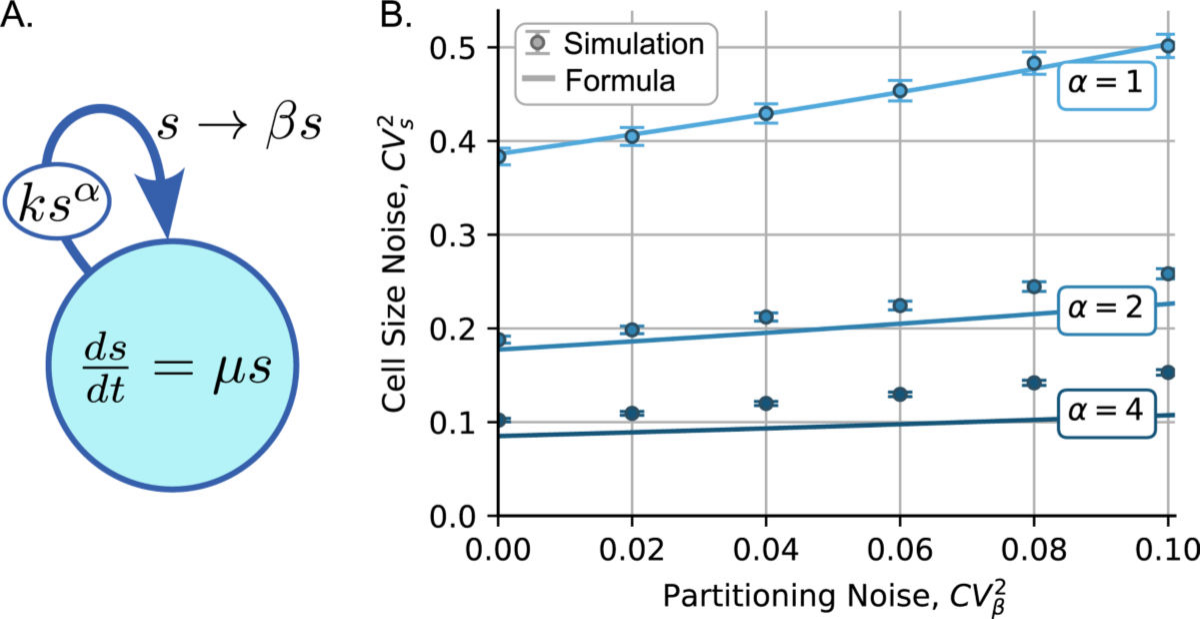
A) SHS schematic with division rate proportional to $s^{\alpha }$ where α>0. B) A comparison of cell size noise approximated by (34c) with exact values obtained from running 10^4^ stochastic simulations. When α=1, Formula (34c) is exact and reduces to (15). $CV_{s}^{2}$ increases with increasing noise in partitioning $CV_{\beta }^{2}$. The formula (34c) underestimates the actual noise level. Solid lines represent the formula (34c) and error bars, 95% confidence intervals calculated from 10^4^ simulations.

Given that 〈log(β)〉/(〈β〉−1)>1, $CV_{s}^{2}$ as approximated by (34c) shows that increasing α will lead to a reduction in cell size variations consistent with exact noise levels obtained from stochastic simulations of the corresponding SHS (Fig. 2).

## IV. Multi-Step Models of Cell Size Control

In the previous section, we investigated the cell size statistics for single-step models, with (21) predicting the expected noise for an adder. Based on experimental evidence from bacterial cells the observed cell size noise can be several folds lower than as predicted by (21) [21], [22]. To address this mismatch, we expand the model by conceptualizing the cell cycle as a progression through a total of M stages. Here, cells are born in stage i=1, and transition between stages i∈{1,2,…,M−1} to i+1 with a rate $k_{i}s$ proportional to size. When reaching stage i=M, the cell divides at a rate $k_{M}s$. Here M=1 recovers the previous single-step SHS model. This model can also be recast as the size-dependent buildup of a cell-cycle regulator, where the stages are the number of regulator molecules and division is triggered upon reaching a critical threshold count M [18].

Using Bernoulli random variables $g_{i}(t)=1\;\left(g_{i}(t)=0\right)$ to denote that the cell is (is not) in cell-cycle state i, the transition rate from stage i to i+1 is given by $k_{i}g_{i}(t)s(t)$, and this transition between stages is captured by the reset

(35) $$g_{i}\mapsto g_{i}-1,\;g_{i+1}\mapsto g_{i+1}+1,\;i\in \{1,2,\ldots ,M-1\}.$$

Finally, the cell division event occurs with rate $k_{M}g_{M}(t)s(t)$, and the corresponding resets capturing the change in stage from M back to 1 and the cell size decrease are

(36) $$g_{M}\mapsto g_{M}-1,\;g_{1}\mapsto g_{1}+1,\;s\mapsto \beta s.$$

Note that $\sum_{i=1}^{M}g_{i}(t)=1$ and the expectation of the product $\langle g_{i}g_{j}\rangle =0$ for i≠j.

### A. Average Cell Size

Taking a similar approach as in the previous section, first writing the moment dynamics of log(s) and solving it at steady-state yields

(37) $$\;\frac{d\langle \log(s)\rangle }{dt}=\mu +k_{M}\langle g_{M}s(\log(\beta s)-\log(s))\rangle \;\Rightarrow \bar{\langle g_{M}s\rangle }=-\frac{\mu }{k_{M}\langle \log(\beta )\rangle }.$$

Similarly, we obtain

(38) $$\bar{\langle g_{i}s\rangle }=-\frac{\mu }{k_{i}\langle \log(\beta )\rangle },\;i\in \{1,2,\ldots ,M\}$$

by considering the moment dynamics of $\langle g_{i}\rangle$ at steady-state leading to the following steady-state mean cell size

(39) $$\bar{\langle s\rangle }=\sum_{i=1}^{M}\bar{\langle g_{i}s\rangle }=-\frac{\mu }{\langle \log(\beta )\rangle }\sum_{i=1}^{M}\frac{1}{k_{i}}.$$

### B. Noise in Cell Size

Using the time evolution of the mean cell size we obtain

(40) $$\frac{d\langle s\rangle }{dt}=\mu s+k_{M}\langle g_{M}s(\beta s-s)\rangle$$

(41) $$\Rightarrow \bar{\langle g_{M}s^{2}\rangle }=\frac{\mu \bar{\langle s\rangle }}{k_{M}(1-\langle \beta \rangle )}.$$

Now leveraging the moment dynamics of $\langle g_{1}s\rangle$ given by

(42) $$\frac{d\langle g_{1}s\rangle }{dt}=\mu \langle g_{1}s\rangle +\langle k_{M}g_{M}s\left(\left(g_{1}+1\right)\beta s-g_{1}s\right)\rangle \;\;+\langle k_{1}g_{1}s\left(\left(g_{1}-1\right)s-g_{1}s\right)\rangle$$

and using (38) and (41) we determine at steady-state

(43) $$\bar{\langle g_{1}s^{2}\rangle }=\frac{\mu }{k_{1}}\langle g_{1}s\rangle +\frac{k_{M}}{k_{1}}\langle \beta \rangle \bar{\langle g_{M}s^{2}\rangle }\;\;=-{\left(\frac{\mu }{k_{1}}\right)}^{2}\frac{1}{\langle \log(\beta )\rangle }+\frac{\mu \bar{\langle s\rangle }\langle \beta \rangle }{k_{1}(1-\langle \beta \rangle )}.$$

Repeating this process iteratively for $\langle g_{i}s\rangle$ results in

(44) $$\bar{\langle g_{i+1}s^{2}\rangle }=-{\left(\frac{\mu }{k_{i+1}}\right)}^{2}\frac{1}{\langle \log(\beta )\rangle }+\frac{k_{i}}{k_{i+1}}\bar{\langle g_{i}s^{2}\rangle }$$

i∈{2,…,M−1} that can then be used to find

(45) $$\bar{\langle s^{2}\rangle }=\sum_{i=1}^{M}\bar{\langle g_{i}s^{2}\rangle }\;\;=\frac{\mu \bar{\langle s\rangle }}{1-\langle \beta \rangle }\sum_{i=1}^{M}\frac{1}{k_{i}}+\frac{\mu^{2}}{\langle \log(\beta )\rangle }\sum_{i=1}^{M}\sum_{j=1(j\neq i)}^{M}\frac{1}{k_{i}k_{j}}.$$

For a fixed $k_{1}$, in the limit $k_{i}\to \infty$, i∈{2,…,M}, (45) reduces to (13).

### C. Equal Transition Rates

In this section, we consider a special case of the model with equal transition rates

(46) $$k_{i}=kM\;i\in \{1,2,\ldots ,M\}$$

where the size added from birth to division is an Erlang distribution with mean μ/k and noise $CV_{\text{Δ}}^{2}=1/M$ [15]. Using $k_{i}=kM$ in (39) and (45) give the following *exact* analytical expressions

(47a) $$\bar{\langle s\rangle }=-\frac{\mu }{k\langle \log(\beta )\rangle }$$

(47b) $$CV_{s}^{2}=-1+\left(\frac{1+M+\langle \beta \rangle (M-1)}{2M}\right)\frac{\langle \log(\beta )\rangle }{\langle \beta \rangle -1}$$

for steady-state mean and noise in cell size, respectively, while from (22) the noise in newborn cell size is

(48) $$CV_{s_{b}}^{2}=\frac{4CV_{\beta }^{2}+\frac{1+CV_{\beta }^{2}}{M}}{3-CV_{\beta }^{2}}.$$

**Fig. 3.**
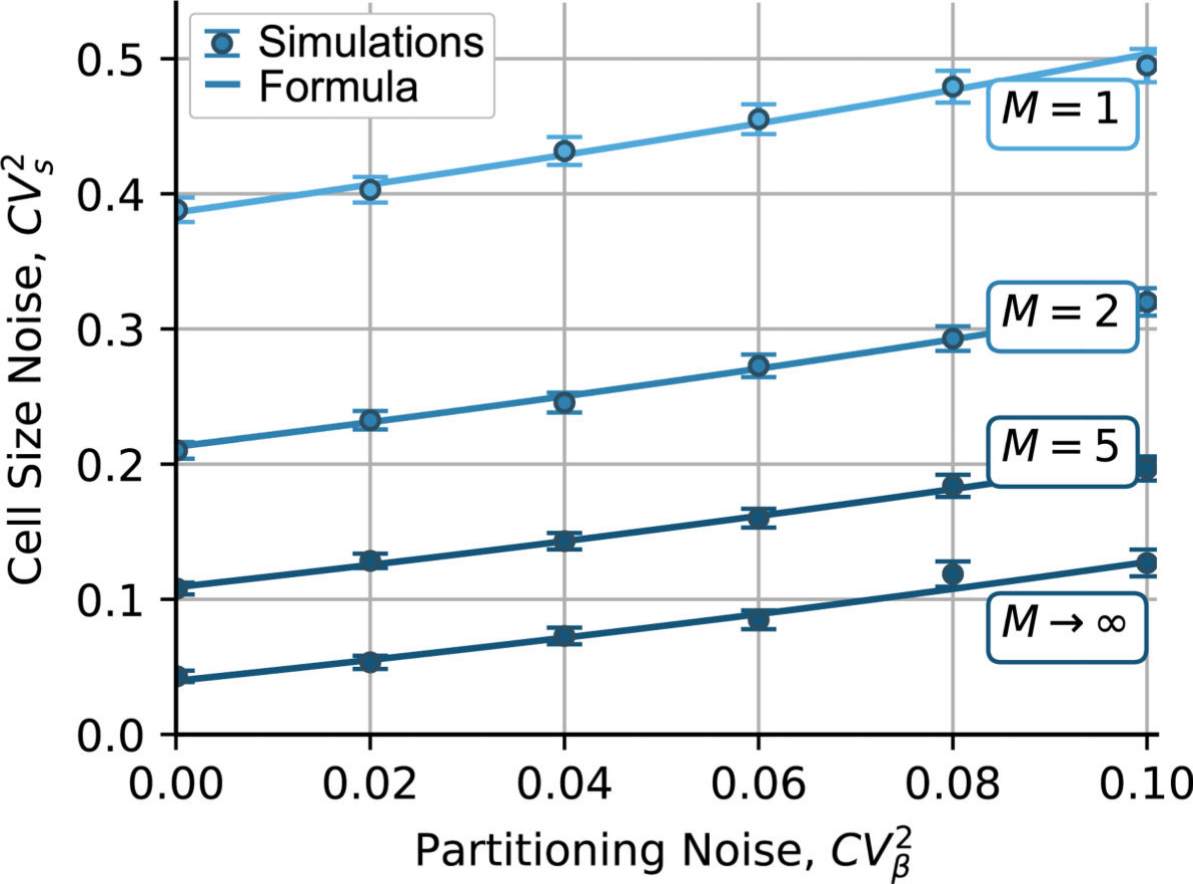
Noise in cell size as predicted by (47b) for the multi-step adder model where newborns transition through stages i∈{1,…,M} before division at rate kMs(t). Steady-state noise in cell size $CV_{s}^{2}$ increases with $CV_{\beta }^{2}$ and decreases with increasing M to its minimum value as M→∞ following (49). Solid lines represent the formula (47b) and error bars, 95% confidence intervals calculated from 10^4^ simulations.

Substituting M=1 in these formulas, we recover our earlier results for a single-step adder. As illustrated in Fig. 3, $CV_{s}^{2}$ monotonically decreases as M increases approaching

(49) $$\underset{M\to \infty }{\lim}CV_{s}^{2}=-1+\left(\frac{1+\langle \beta \rangle }{2}\right)\frac{\langle \log(\beta )\rangle }{\langle \beta \rangle -1}.$$

Deploying the approximation of 〈log(β)〉 in (20) with 〈β〉=1/2, $CV_{s}^{2}$ can be decomposed as a sum of two terms

(50) $$CV_{s}^{2}\approx -1+\frac{\log(8)}{2}+\frac{\log(2)}{2M}+\frac{CV_{\beta }^{2}(1+3M)}{4M\left(1-CV_{\beta }^{2}\right)}$$

assuming $CV_{\beta }^{2}\ll 1$. The first-term (50) is the noise level for perfect symmetric partitioning $\left(CV_{\beta }^{2}=0\right)$) that varies from ≈ 0.39 (M=1) to ≈ 0.04 (M→∞) – a ten-fold decrease in noise levels. While the second term (50) is the contribution of partitioning errors varying from

(51) $$\frac{CV_{\beta }^{2}}{\left(1-CV_{\beta }^{2}\right)}\;\text{for}\;M=1$$

to decreasing to 3/4 of its value

(52) $$\frac{3CV_{\beta }^{2}}{4\left(1-CV_{\beta }^{2}\right)}\;\text{for}\;M\to \infty .$$

## V. Conclusion

Modeling the stochastic dynamics of cell size, this letter derives exact steady-state mean, coefficient of ariation squared (noise) and skewness of cell size when cells divide at a rate proportional to their size. In this simple one-step adder model, cell size noise depends solely on the parameter β∈(0,1). For perfect symmetric partitioning (β=1/2 with probability one), $CV_{s}^{2}$ equals log(4) − 1 $\left(CV_{s}\approx 0.63\right)$, implying an approximately 63% variation in size around the mean. The formulas reveal that $CV_{s}^{2}$ is a monotonically decreasing function of the average value of β and a monotonically increasing function of noise in $\beta \;\left(CV_{\beta }^{2}\right)$. Furthermore, we extend these results to consider a generalized division rate $ks^{\alpha }$ with a reduction in cell size variations with increasing α (Fig. 2).

The single-step adder discussed above predicts cell size variations that are much higher than those observed in physiological cell populations. This motivates the multistep adder, where the cell cycle is divided into M stages, and stage transitions occur at a rate proportional to size. Our results provide an exact expression of the mean and noise in cell size for this multi-step model, with noise decreasing with increasing number of stages (Fig. 3). These formulas reveal the fundamental limit to which cell size noise can be reduced for large M that is given by

(53) $$\underset{M\to \infty }{\lim}CV_{s}^{2}\approx 0.04+\frac{3CV_{\beta }^{2}}{4},\;CV_{\beta }^{2}\ll 1.$$

Although our current focus has been on steady-state moments, we aim to generalize these findings to capture the time evolution of moments. By tracking individual cell size over time, we can infer the underlying processes governing cell size control. Examples of this kind of inference include several works [14], [15], [22], [23], where combining model-predicted cell size statistics with single-cell data gives insight into hidden variables such as the number of division stages M, the form of stage transition rates, and how intercellular variability in cell size can be attenuated by regulating these processes. These models can be generalized to consider diverse division rates leading to more complex forms of size control. For example, in fission yeast, where while small/medium-sized newborns follow the sizer, larger newborns follow the adder [24]. Furthermore, the SHS model can be modified to capture size control in the green alga *Chlamydomonas reinhardtii* where a mother cell gives rise to $2^{n}$ daughters, where n∈{1,2,…} is size dependent [25].
